# The Unique Biology behind the Early Onset of Breast Cancer

**DOI:** 10.3390/genes12030372

**Published:** 2021-03-05

**Authors:** Alaa Siddig, Tengku Ahmad Damitri Al-Astani Tengku Din, Siti Norasikin Mohd Nafi, Maya Mazuwin Yahya, Sarina Sulong, Wan Faiziah Wan Abdul Rahman

**Affiliations:** 1 Department of Pathology, School of Medical Sciences, Health Campus, Universiti Sains Malaysia, Kelantan 16150, Malaysia; alaaabdelaziz@student.usm.my (A.S.); snmn@usm.my (S.N.M.N.); 2 Department of Chemical Pathology, School of Medical Sciences, Health Campus, Universiti Sains Malaysia, Kelantan 16150, Malaysia; damitri@usm.my; 3 Breast Cancer Awareness & Research Unit, Hospital Universiti Sains Malaysia, Kelantan 16150, Malaysia; mazuwin@usm.my; 4 Department of Surgery, School of Medical Sciences, Health Campus, Universiti Sains Malaysia, Kelantan 16150, Malaysia; 5 Human Genome Centre, School of Medical Sciences, Health Campus, Universiti Sains Malaysia, Kelantan 16150, Malaysia; ssarina@usm.my

**Keywords:** early-onset breast cancer, differential gene expression, somatic mutations, breast cancer in young age, copy number variants, gene methylation, extracellular matrix protein-coding genes

## Abstract

Breast cancer commonly affects women of older age; however, in developing countries, up to 20% of breast cancer cases present in young women (younger than 40 years as defined by oncology literature). Breast cancer in young women is often defined to be aggressive in nature, usually of high histological grade at the time of diagnosis and negative for endocrine receptors with poor overall survival rate. Several researchers have attributed this aggressive nature to a hidden unique biology. However, findings in this aspect remain controversial. Thus, in this article, we aimed to review published work addressing somatic mutations, chromosome copy number variants, single nucleotide polymorphisms, differential gene expression, microRNAs and gene methylation profile of early-onset breast cancer, as well as its altered pathways resulting from those aberrations. Distinct biology behind early-onset of breast cancer was clear among estrogen receptor-positive and sporadic cases. However, further research is needed to determine and validate specific novel markers, which may help in customizing therapy for this group of patients.

## 1. Introduction

Breast cancer has been identified as the most common cancer among women worldwide with an estimated two million new cancer cases diagnosed in 2018, accounting for 23% of all cancer types [1]. Breast cancer is a disease characterized by having diverse clinical behaviors and different biological characteristics, making the process of prediction and management more challenging for physicians, breast surgeons, and oncologists [2]. Advancement in molecular technologies revealed that breast cancer is not a single disease, but is a group of conditions with distinct molecular profiles [3]. Predominantly, breast cancer affects women older than 40 years. Yet, in some parts of the world such as in Eastern Asia, the Middle East, North Africa, and South America, breast cancer in young women (<40 years) had high frequencies [4]. Incidence of early-onset breast cancer (EOBC) was estimated to reach 6%–10% of all breast cancer cases in developed countries; this figure doubled in developing countries where the percentage reaches 20%; the same goes for its mortality rate, that is, 7% vs. 14% for developed and developing countries, respectively [5].

Breast cancer in young women has been defined by its aggressive nature; it tends to be of high histological grade at diagnosis, high proliferation rate, and is positive for human epidermal growth factor receptor (HER-2) and negative for endocrine receptors. In addition, high rate of local recurrence was associated with EOBC [6,7,8,9,10,11,12,13,14]. Kataoka A. et al. found that after adjusting several prognostic factors including nodal status, adjuvant therapy, and breast cancer subtype, young age remained an independent negative prognostic factor for poor prognosis for all three endpoints: 5-year disease-free survival, breast cancer-specific survival, and overall survival [15]. Many researchers assumed that EOBC has distinct biology different from that in late-onset. In their view, this distinction is not only observed in the aggressive phenotype mentioned above, but also in the different distributions of risk factors and the contradictory clinical outcome of patients with comparable clinicopathological parameters and similar therapeutic approach but the only difference is that they belong to different age groups [16,17].

In this article, we reviewed published literature addressing EOBC-related somatic mutations, chromosome copy number variants, single nucleotide polymorphism, differential gene expression profile, microRNAs, DNA methylation profile, and differentially expressed proteins, as well as altered pathways that have resulted from those aberrations. We believe that better understanding of EOBC biology may help in the identification and verification of molecular markers, which is a step towards personalizing therapy for this group of patients who show insufficient efficacy to conventional adjuvant hormone and chemotherapy.

## 2. Contribution of Common Breast Cancer Mutations

Germline mutations acquired in DNA repair and tumor suppressor genes are the most common form of breast cancer genetic susceptibility, which ultimately lead to the accumulation of mutations in cell cycle check point and oncogenes that are required for aberrant cell division [18]. Around 10%–20% of EOBC cases are hereditary [19] *BRCA1* and *BRCA2* are the most common mutated genes related to breast cancer since their discovery in the early 1990s [20]. Mutations related to the development of cancers are often classified as high, intermediate, and low penetrance mutation based on their relative risk for the specific cancer. *BRCA1*, *BRCA2*, *TP53*, *PTEN*, *STK11*, and *CDH1* are considered the high penetrance mutations of breast cancer where they account for 20% of hereditary risk. This is followed by moderate penetrance mutations, which include *PALB2*, *BRIP1*, *ATM*, *CHEK2*, and *RAD51C*, which account for around 5% of hereditary risk [21]. In addition, more than 180 mutations are considered as low-risk loci for breast cancer, which explains only 18% of the familial risk. All these relative risk proportions define only half of the genetic risk of breast cancer, with the other half still unknown [22]. In this study, we found that the relative risk of different mutations differs among different age groups. In Table 1. we provide a summary of the contributions of common breast cancer mutations to EOBC.

## 3. Prevalence of *BRCA1* and *BRCA2* Mutations

*BRCA1* mutations were attributed to the high frequency of triple-negative and high-grade tumors seen in EOBC [24]. However, the frequency of *BRCA1* mutations among different populations has been found to vary widely (5.9%, 22.7%, 12%, 12.2%, and 6.2% among British, Italian, American, Polish, and Chinese, respectively) [25,26,27,28,29], and still aggressive characteristics of EOBC persist. Conversely, *BRCA2* mutations show less contribution to EOBC, and, even if detected, it does not appear to correlate with the aggressive phenotype (high-grade, negative estrogen and progesterone receptor, high proliferation rate). Thus, in-depth investigation should be conducted among heterogeneous EOBC cohort to reveal the shared mutational pattern and frequency of *BRCA1*.

## 4. Somatic Mutations

In 2017, Bryan et al. proved that no germline mutations were associated with the mortality rate and aggressive nature of EOBC [30], directing later research towards somatic and transcriptomic variations. Using The Cancer Genome Atlas (TCGA) and Molecular Taxonomy of Breast Cancer International Consortium (METABRIC) databases, young age estrogen receptor-positive breast cancer patients (≤45 years) showed high mutational rate in *CDH1* gene in comparison to older age patients (≥55 years) (14.5% vs. 2.9%). It should be noted that *CDH1* mutations are common findings in lobular breast carcinoma, which mostly affects older age patients. However, in this study, *CDH1* mutations were found to be significantly high among young age patients even after amendment for multiple comparisons [31]. This may suggest a shared mutational profile between lobular carcinoma and EOBC. *CDH1* gene encodes E-cadherin protein, which plays a significant role in cell-cell adhesion as well as cell-extracellular matrix adhesion [32]. Disturbed expression of E-cadherin was found to be associated with high-grade tumor, metastasis, and poor prognosis [33].

Meanwhile, another two groups of researchers used TCGA public database to determine the landscape of somatic mutations in EOBC. The first group of researchers found that *GATA3* mutations were the only somatic mutations independently associated with young age patients (<45 years) compared to older age patients (>46 years) even after adjustment of several clinicopathological parameters [34]. The second group reported high proportion of mutations in *GATA3* and *CTNNB1* genes in EOBC patients (≤40 years) compared to late-onset patients (>40 years) [35]. Understanding the role of *GATA3* and *CTNNB1* genes may help in recognizing their contribution to EOBC. In addition to the main role of *GATA3* in mammary gland development [36], *GATA3* is also known to encode a transcription factor involved in T-cell differentiation [37]. Tumors with low GATA binding protein 3 (GATA3) expression levels were found to be associated with shorter overall and disease-free survival compared to tumors with high GATA3 expression levels [38]. In summary, *GATA3* mutations contribute to cancer initiation, resistance to endocrine therapy, and poor prognosis [39]. On the other hand, *CTNNB1* gene encodes β-catenin protein, which is an important protein for intercellular structure and cell growth modulation. β-Catenin has been determined to be vital in cell-cell adhesion. Disturbance in β-catenin expression may result in tumor metastasis [40].

In a recent study that examined 90 Taiwanese women with EOBC (<41 years), whole genome sequencing and whole exon sequencing revealed that 40% and 37% of patients were harboring mutations in tumor suppressor gene *TP53* and oncogene *PIK3CA*, respectively. The unexpected findings were the high frequency of extracellular structural protein-coding gene mutations *MUC17* (19%), *TTN* (17%), and *FLG* (16%). Comparing the frequencies of the three mutations between non-Taiwanese pooled EOBC and pooled non-EOBC cohorts, *MUC17* and *FLG* mutations were found to remain distinctly high [41]. Little is known about *MUC17* implications for breast cancer. However, it is important to note that *MUC1* and *MUC4* have been reported to induce chemoresistance, possibly through creating physical barrier minimizing tumoral drug concentration or by reducing apoptosis [42]. Al Amri et al. presented *MUC17* expression as chemotherapy predictive markers in breast cancer; further, in vitro knockdown of *MUC17* was associated with enhanced chemotherapy sensitivity. Moreover, survival analysis showed that low level of *MUC17* expression was related to longer survival following chemotherapy [43]. On the other hand, protein encoded by Filaggrin (*FLG*) is identified as an intermediate filament-associated protein that aggregates keratin intermediate filaments in mammalian epidermis, which, in turn, promotes epidermal barrier formation [44]. Thus, it was suggested that mutations in *FLG* might disturb the physical barrier, increasing the permeability of environmental carcinogens resulting in somatic mutations and tumorigenesis at an early age [41].

There is more evidence to support that EOBC somatic mutation profile is different from that of late-onset breast cancer. In a study that examined young Latin American breast cancer patients, *TP53* mutations showed an unexpected pattern of mutations not common in breast cancer, where 27% of the mutations were single-base substitution-transversion mutations G:C > T:A compared to other young and old breast cancer cohorts used for comparison (METABRIC, TCGA, and International Agency for Research on Cancer IARC) [45]. This proportion and pattern of mutations were described earlier in lung cancer patients and attributed to exposure to exogenous agent, polycyclic aromatic hydrocarbons (PAHs) [46]. Surprisingly, studies in the field of occupational and environmental medicine provided evidence of increased risk of breast cancer related to exposure to PAHs, mainly among cases with family history and premenopausal status [47,48]. This association needs further investigation to understand why carcinogenic PAHs develop EOBC more than late-onset breast cancer.

To summarize, although the cut-off age of EOBC differs from one study to another, as varying public databases were also used to generate these results, we have realized that most studies examining somatic mutations share the same mutational theme, that is, the higher mutational frequency in genes encodes adhesion and extracellular matrix molecules. This is consistent with EOBC infiltrating and metastatic behaviors, considering the important role of the extracellular matrix (ECM) in providing structural and biochemical support for cancer stem cells that in turn induces cancer stemness [49]. Inhibitors that target ECM can, therefore, be an effective choice in the treatment of EOBC patients. However, more research in this area is needed. Additionally, genetic panel testing of most frequent mutations (*TP53*, *CDH1*, *PIK3CA*, *GATA3*, and *CTNNB1*) can assist in EOBC management and prediction of tumor prognosis. The ethnicity of the patient, however, should also be taken into consideration, as it is apparent that the incidence of mutations varies between different ethnicities.

## 5. Chromosome Copy Number Variants (CNVs)

Chromosome copy number variant (CNV) is a form of genetic structural variation, defined by any increase or decrease in the number of DNA segments that measure around one kilobase (1000 base pairs) or more [50]. Azim et al. detected one chromosome copy number variant to be significantly associated with EOBC (≤45 years) (deletion in chr6q27) in comparison to late-onset breast cancer (≥70 years) [34]. Investigating the significance of genes located in chromosome 6, it appears that chromosome 6 long arm holds genes that reflects tumorigenic and metastatic features in many cancer cell lines [51,52], in particular, growth-suppressing genes in breast cancer cells. The significance of allele loss in chromosome 6 to breast cancer is due to the localization of estrogen receptor in 6q23–6q25 regions; loss in these regions may give rise to less differentiated and aggressive breast tumors [52].

Another group of researchers using Genomic Identification of Significant Targets (GISTIC) tool recognized two regions of amplification (6p23 and 7p21.1) and three regions of deletion (19q13.32, 22q12.3, and 22q13.31) in estrogen receptor-positive breast cancer young age patients (≤45 years) compared to their older counterparts (≥55 years) [31]. To emphasize the impact of those chromosomal aberrations on breast cancer, loss and gain in chromosome 7 short arm were reported in primary breast cancer and early-stage lung adenocarcinoma [53,54]; genes present within chromosome 7 were suggested to play an important role in breast cancer tumorigenesis [53]. Loss of heterozygosity (LOH) in chromosome 19q13.3 was detected with higher frequency in secondary glioblastomas compared to primary glioblastomas, providing evidence that LOH in 19q13.3 may contribute to disease progression. An additional point to note is that chromosome 19q13.3 region contains *BAX* gene [55], which induces cell apoptosis. Disturbed apoptotic pathways resulted in cancer growth and resistance to anti-cancer therapy. Low expression of *BAX* gene was reported in all breast cancer subtypes [56,57]. Lastly, loss of chromosome 22 long arm is common in breast cancer. Castells et al. reported loss in chromosome 22q in more than half of invasive ductal carcinoma cases (15/28) and 75% of lobular carcinoma cases (3/4); conversely, none of the ductal carcinomas in situ showed loss in any 22q loci. Thus, it was assumed that the long arm of chromosome 22 contains genes that might have contributed to advance-stage tumor progression [58].

Meanwhile, Ghaffari et al. reported a change in copy number variant in *BIRC5* gene among EOBC patients, out of 40 breast cancer tissue samples tested, 7 samples showed amplification in *BIRC5*, of which 5 were from patients younger than 40 years at diagnosis [59]. *BIRC5* gene contributes to apoptosis modulation and interleukin-11 signaling pathway. Thus, it may play an important role in cell cycle regulation and various cell cycle checkpoints [60]. A previous study reported correlation between *BIRC5* expression and increased survival time to relapse or death [61].

To conclude, EOBC copy number variant-based studies presented CNVs contributed to EOBC tumorigenesis (6q27, 6p32, and 7p21.1), advance-stage tumor progression (22q12.3 and 22q13.31), disease progression (19q13.32), and prognosis (copy number variant in BIRC5 gene). However, further studies that correlate CNV profile with gene and protein expression profile are needed to validate these findings.

## 6. Single Nucleotide Polymorphism

Single nucleotide polymorphism (SNP) is a form of genetic variation, which ultimately may affect protein structure and function [62]. An interesting meta-analysis study that examined 6042 breast cancer patients, including 2315 aged ≤ 40 years at diagnosis, identified 2 SNPs associated with disease progression in EOBC (≤40years). Both SNP rs715212 (P meta = 3.54 × 10^−5^) and rs10963755 (P meta = 3.91 × 10^−4^) were found in the *ADAMTSL1* gene. Further expression quantitative trait locus analysis supports the hypothesis that rs715212 may influence Amphiregulin (*AREG*) expression [7]. *AREG* gene, which shows higher expression in EOBC [31], has been determined to regulate T-regulatory cells creating an immune-suppressed tumor environment [63]; most importantly, it contributes to resistance to chemotherapy [64].

Meanwhile, a comparable study that examined only Caucasian women detected 12 SNPs associated with EOBC. All these 12 SNPs were near or within the *MAP3K1* gene. The SNPs with lowest *p*-value were rs2229882 and rs889312. However, after multiple comparisons, most of the SNPs were also detected in late-onset breast cancer, which made the researchers suggest that late- and EOBC may share the same genetic profile [65]. Unexpectedly, a subsequent study found that SNP, MAP3K1 rs889312 (C/C allele) was significantly associated with poor disease-free survival, distant disease-free survival, and overall survival in hormone receptor-positive breast cancer patients, predominantly in premenopausal patients [66].

Tunisian EOBC patients (<40 years) exhibited genetic polymorphism of major histocompatibility complex class I-related chain A (MICA) in comparison to their older counterparts. MICA is a glycoprotein that has been determined to play an important role in the modulation of host immune response, suggesting that it may mediate cell viability by letting tumor cell evade host immune system [67]. 

Considering the above findings, SNP studies highlighted the significant contributions of different SNPs to EOBC tumorigenesis, progression, resistance to chemotherapy, and poor prognosis. However, most of those studies were limited to homogenous populations. Thus, larger sample size studies with heterogeneous population will be needed to validate and generalize these findings.

## 7. Differential Gene Expression Profile

In 2008, Andres and her colleagues found that gene occurrence in breast cancer patients appears in an age-related manner; further, they determined that there are more than 367 gene sets differentially expressed in young patients (≤45 years) in comparison to older patients (≥65 years). However, in 2011, the same group of researchers reanalyzed the exact data by building a statistical model to demonstrate differentially expressed genes between the two age groups and to exclude any cofounders. The model yielded 693 genes, but after clinicopathological correlation, gene expression differences diminished to 0 [68]. In contrast, a literature-based study revealed that breast cancer in young women (≤40 years) was differentially enriched with gene sets representing luminal progenitor cells, immature mammary stem cells, and high levels of *RANKL*, *c-kit*, and *BRCA1* mutations [69]. These controversial findings challenged more researchers to further investigate in this area.

Liao et al. reported 178 genes differentially expressed in young age estrogen receptor-positive breast cancer patients (≤45 years) compared to their older counterparts (≥55 years). In young age patients, significant genes were upregulated such as *AREG*, *TFPI2*, *AMPH*, *DBX2*, *RP5-1054A22.3*, and *KLK5*, while *ESR1*, *CYP4Z1*, *RANBP3L*, *FOXD2*, and *PEX3* were downregulated [31]. Yau C. et al. have also reported 24 genes highly expressed in estrogen receptor-positive sporadic breast cancer young age patients (≤45 years) (*AREG*, *PRSS*, *GREB*, *PTHLH*, *HPGD*, *STK6*, *FGFR1*, and *DLG7*) compared to old age patients (≥70 years) [70]. Implications of up- and downregulated genes to breast cancer are summarized in Table 2.

Analyses of two microarray datasets (GSE109169-GSE89116) have revealed differential expression of *PPARG* and *SQLE* genes in EOBC tumor tissues (<40 years) compared to normal breast tissues. *PPARG* was downregulated in EOBC tumor tissue [87]. It was established that the level of Peroxisome proliferator-activated receptor γ (PPARG) is related to breast cancer patient prognosis. Low level of expression of PPARG was observed in patients with local recurrence and in patients who died of breast cancer [88]. Meanwhile, *SQLE*, which encodes squalene epoxidase a rate-limiting enzyme in sterol biosynthesis and a significant therapeutic target for breast cancer, was upregulated in EOBC tumor tissue [87]. *SQLE* expression, which was associated with poor prognosis, was found to be highly expressed among black women compared to white women in a previous study [89]. Taking into account that black women have twice the incidence of EOBC [10], and by considering that this study used data generated from two Asian population (Indian and Taiwanese), we assumed that *SQLE* expression in breast cancer is related to EOBC more than patient ethnicity.

In conclusion, it can be noted that most EOBC gene expression studies include only estrogen receptor-positive breast cancer cases, as there is lacking difference in gene expression profile of estrogen receptor-negative cases among different age groups. However, the findings of those studies are valuable and serves as guidance for further investigation.

## 8. MicroRNAs

MicroRNAs (miRNAs) are small RNA molecules that regulate gene expression post-transcriptionally. MicroRNAs are often considered new biomarkers utilized in the prediction of various biological processes, such as cell differentiation, cell cycle regulation, development, and apoptosis. Any disruption in the microRNA expression may result in a change in the gene expression profile of the cell and, consequently, in initiation and progression of syndromes, including cancer [90].

It is possible to discriminate breast cancer arising in young women from that in older women using microRNA profile. As per the findings of Peña-Chilet et al., it was determined that normal breast tissue and breast cancer tissue from older women (≥65 years) share almost similar microRNA profile, contrary to the profile obtained from younger women below the age of 35 years [91].

Several microRNAs were implicated in the aggressive phenotype seen in EOBC; eight microRNAs were differentially expressed in estrogen receptor-positive breast tumors from young age patients (35 years or less) compared to tumors from older patients (50–65 years). Seven of these microRNAs were upregulated (miR-9, miR-210, miR-106a, miR-106b, miR-18b, miR-33b, and miR-518a-3p), and only one was downregulated (miR-372). The expression level of most of those deregulated microRNAs was associated with higher tumor size and TNM stage (*p*-value < 0.05) [92].

Breast tumors from very young age patients (35 years or less) showed differential expression of six microRNAs (hsa-miR-1228*, hsa-miR-3196, hsa-miR-1275, hsa-miR-1207-5p, hsa-miR-92b and hsa-miR-139-5p) compared to tumors from patients above 65 years of age. The deregulated microRNAs were significant for pathways relevant to apoptosis, cell motility, proliferation, mitotic regulatory processes, phosphatidylinositol 3-kinase (PI3K) and Insulin–like growth factor-1 receptor (IGFR) transduction [91]; all those pathways grant for the tumor high metastatic capacity by inducing progression and invasion.

Three microRNA expressions (miR-1285-5p, miR-183-5p, and miR-194-5p) were determined to be correlated with overall survival of very young breast cancer patients (<35 years) who had poor prognosis (recurrence within 5 years of primary diagnosis) [4]. Differentially expressed miRNAs serve as a promising area for further exploration. Implications of all deregulated microRNAs in EOBC mentioned in this section is described in Table 3.

## 9. DNA Methylation Profile

DNA methylation is one of the epigenetic machineries deemed crucial for the normal development and maintenance of tissue-specific gene expression [115]. EOBC has been often characterized by having hypomethylated DNA profile in comparison to late-onset breast cancer, where hypomethylation was determined in 69% of significant CpG sites. Pathways affected by methylation in EOBC include those related to the neuronal system, extracellular matrix modulation, immune system, DNA repair, Notch/Notch1 signaling, and vesicular trafficking. DNA methylation in EOBC resulted in significant upregulation of *HDAC5* which proved to promote tamoxifen resistance through cancer stem cell-related transcription factor SOX9 deacetylation, as well as significant downregulation of *EHF* that involves in epithelial mesenchymal transition inducing metastasis [116].

By using TCGA methylation data, 373 genes were hypomethylated, whereas 457 genes were hypermethylated in estrogen receptor-positive young age breast cancer patients (≤45 years) compared to their older counterparts (≥65 years). The significant hypermethylated genes were *ESR1*, *MAT2B*, *CTSS*, *DDR2*, and *GALNTL2* [31]. Methionine Adenosyltransferase 2B (*MAT2B*) has been determined to be involved in cell metabolism including proliferation and apoptosis; further, higher expression of *MAT2B* was correlated with good prognosis in estrogen receptor-positive breast cancer patients [117]. Meanwhile, *CTSS* gene has been identified to encode Cathepsin S protein which plays a significant role in genomic stability through abolishing BRCA1 activity; further, *CTTS* knockdown was found to be associated with suppression of tumor metastasis in triple-negative breast cancer cell [118]. Overall, DNA methylation events appears to play vital role in EOBC aggressive characteristics.

## 10. Differentially Expressed Proteins

Several proteins showed significant differential expression in young age patients (less than 35 years) compared to older age patients (between 50 and 65 years). Bcl-2-like protein 1 (BCL2L1), Poly [ADP-ribose] polymerase 1 (PRP1), and RAF proto-oncogene serine (RAF1) were determined to be overexpressed, whereas Estrogen receptor (ESR1), Eukaryotic translation initiation factor 4E (EIF4E), Signal transducer and activator of transcription 5A (STAT5A), and Ribosomal protein S6 kinase alpha-1 (RPS6KA1) were underexpressed. The pattern of expression of the deregulated proteins was linked to the clinical parameters of EOBC. Higher expression of PRP1 and lower expression of RPS6KA1 were associated with positive lymph nodes status, whereas overexpression of RAF1 and underexpression of STAT5A were correlated with high TNM stage [92].

By utilizing immunohistochemical staining, Hasoda et al. recognized significant higher expression level of Receptor activator of nuclear factor kappa-Β ligand (RANKL), GATA3, Progesterone receptor (PgR), and Trefoil factor 1 (TFF1) in estrogen-positive and HER-2-negative breast cancer patients (age range between 27 and 56 years) [119]. Table 4. illustrates the functions of over- and underexpressed proteins in breast cancer.

## 11. Altered Pathways

The proto-oncogene MYC signaling pathway has been found to be significantly expressed in EOBC (≤45 years) compared to late-onset breast cancer (≥45 years) [129]. MYC pathway deregulation has been determined to play a role in the development, progression, metastasis, and therapy resistance of breast cancer [130]. Expression of MYC pathway can vary among different molecular subtypes of breast cancer. However, it is often overexpressed in aggressive subtypes such as basal-like subtype [130]. Furthermore, as EOBC has been determined to be rich in basal-like subtype, it was expected that MYC signaling pathways show higher expression. 

Integrin, laminin, and epidermal growth factor receptor signaling pathways were the most significant altered pathways in young age estrogen receptor-positive breast cancer patients (≤45 years) compared to older age patients [31]. Integrin membrane proteins maintain cell adhesion to the extracellular matrix, and upon loss of integrin, the cells will undergo apoptosis. Thus, integrin expression then mediates cell survival and is one of the factors that prevent the tumor cells from undergoing drug-induced apoptosis. β1 integrin was also reported to mediate pathways that drive resistance to HER2-targeted therapies [131,132]. Integrin plays an important role in the migration, proliferation, and death of breast cancer cells [133]. A crosstalk between P-cadherin and laminin receptor and α 6β4 integrin signaling pathway was reported in stem cell and invasive properties of breast cancer cells [134]. On the other hand, epidermal growth factor receptor signaling pathway is implicated in tamoxifen resistance, through activation of downstream kinases, extracellular signal-regulated protein kinases 1 and 2 (ERK1/2), MAP kinase (MAPK) and protein kinase B (AKT) [135]. Thus, it is clear that EOBC exhibits differential expression of pathways related to HER2-targeted therapies and induced tamoxifen resistance. This may provide a clue for tumor recurrence and poor outcome observed in EOBC.

## 12. Tumor Microenvironment

Tumor microenvironment plays an important role in breast cancer tumorigenesis and progression. Targeting malignant and non-malignant components of tumor microenvironment may help in cancer management [136]. Researchers suggested that endocrine changes during the reproductive age and gestation play a critical role in altering breast microenvironment in young women, as if it predisposes the tissue for tumorigenesis [137]. Eight stromal genes were differentially expressed in breast tumors from very young patients (35 years or less) compared to tumors from older age patients (50–65 years) (*UQCRQ*, *ALDH1A3*, *EGLN1*, and *IGF1* overexpressed, while *FUT9*, *IDI2*, *PDHX*, and *CCL18* underexpressed) [92]. Table 5 provides a summary of the implications of EOBC-deregulated stromal genes in breast cancer.

## 13. TP53

It is impossible to skip the role of *TP53* when dealing with EOBC. Thus, in here, we have also mentioned a recent and interesting study that reported significant lower expression of growth-arrest-specific 7 isoform b (*GAS7b*) in young age breast cancer patients (≤40 years old) compared to their older counterparts. Importantly, it should be noted that in normal physiological condition, wildtype *TP53* binds to *GAS7b* promoter, inducing *GAS7b* transcription. However, in EOBC, the high mutational load of *TP53* affects the rate of *GAS7b* transcription; considering the role of *GAS7b* in regulating cell structure and cell migration, this aberrant transcription may contribute to metastasis events [146]. This study revealed that *TP53* mutations may have more implications beyond what was reported earlier; thus, further research is needed.

## 14. Conclusions

Considering all the above evidence, it can be concluded that EOBC has a distinct biology; however, this distinction is more prominent among estrogen receptor-positive and sporadic breast cancer tumors. Further advanced research is needed in order to discover novel molecular markers associated exclusively with EOBC, which may help in customizing the therapy for this group of patients. Additionally, genes related to tumor microenvironment and extracellular matrix proteins, in addition to pathways affected by TP53 mutations, may be a promising area for future research.

## Figures and Tables

**Table 1:** Contribution of common breast cancer mutations to early-onset breast cancer (EOBC).

Gene Penetrance	Gene	Encoded Protein	Significance of Encoded Protein	Condition(s) Related to Gene Mutations	Risk of Breast Cancer Compared to Normal Population	Relative Risk for Breast Cancer Patients Younger than 40 Years Old
High penetrance	*BRCA1*	Nuclear phosphoprotein	DNA damage response, centrosome number, cell cycle progression, and transcriptional regulation	Hereditary breast and ovarian cancer syndrome (HBOC)	40%–80%	9.4%–12%
High penetrance	*BRCA2*	Breast cancer type 2 susceptibility protein (BRCA2)	Repairs DNA breaks during homologous recombination	Hereditary breast and ovarian cancer syndrome (HBOC)	20%–85%	9.4%–12%
Moderate penetrance	*PALB2*	Partner and localizer of BRCA2 (PALB2) protein	Facilitates the co-localization of BRCA2 to DNA damage sites	Fanconi anemia subtype FA-N-Hereditary breast cancer	9.7 times higher than normal population	8–9 times higher than average in patients within 20–39-year age group
Moderate penetrance	*BRIP1*	BRCA1 interacting protein C-terminal helicase 1	Involved in DNA break repair	Fanconi-anemia complementation group J	2%	1%–2%
High penetrance	*TP53*	Tumor protein p53	Tumor suppresser gene	Li-Fraumeni syndrome	Up to 50%	1%–7% in patients with age younger than 35 years
High penetrance	*PTEN*	Phosphatase and tensin homolog	Suppresses the PI3 K/Akt/mTOR pathways and regulates cell survival, proliferation and metabolism	PTEN hamartoma tumour syndrome (PHTS)	30%–50% to 85% in other references	Was reported in literature that women less than 40 years may not harbor *PTEN* mutation
High penetrance	*STK11*/*LKB1*	Serine/threonine kinase 11	Tumor suppressor gene	Peutz-Jeghers syndrome	Up to 45%	8% risk at age of 40 years. In another study, risk to develop breast cancer at 40 years may elevate to 31%
Moderate penetrance	*ATM*	Serine-threonine protein kinase	Identify DNA damage, DNA repair machinery activation, and cell cycle check points	Ataxia-telangiectasia (AT)	Five- to eightfold increased risk for developing breast cancer	Previous studies claimed that *ATM* mutations do not confer genetic predisposition for EOBC
Moderate penetrance	*CHEK2*	G2 checkpoint serine threonine kinase 2	Prevent mitosis when there is DNA damage and replication block	/	Three to five times in patients harbouring the mutation	Heterozygotes of *CHEK2* patients have OR of 2.6 (95% CI, 1.3–5.5) to develop early-onset BC
Low to moderate penetrance	*BARD1*	A protein which interacts with BRCA1 at the N-terminal region	Formation of stable complex with breast cancer type 1 susceptibility protein (BRCA1) which is essential for BRCA1 role as tumor suppresser	/	/	Two- to five-fold
Moderate penetrance	*MRE11*, *RAD50* and *NBN*	MRN complex, which is dimers of three proteins	Involved in DNA repair and cell cycle check points	Nijmegen breakage syndrome and ataxia-telangiectasia-like disorder	Threefold increase for breast cancer risk among heterozygous females	Odds ratio: 2.88 for early-onset breast cancer

(/) not mentioned in literature, relative risk table was summarized from the following references [5,21,23].

**Table 2:** Implications of the upregulated and downregulated genes in EOBC to breast cancer.

GENE	Gene Status in EOBC	Gene Role	References
*AREG*	Upregulated in young age estrogen receptor-positive breast cancer patients (≤45 years)	Amphiregulin (AREG) is a growth factor essential for the development of normal mammary gland and mammary stem cell differentiation as well as estrogen receptor-positive breast tumour. *AREG* was reported to engage in regulating proliferation and migration of HER2-positive breast cancer cells. In vitro study showed that *AREG* knockdown may slow down breast cancer cell growth but will not completely inhibit it. However, it will affect breast cancer cell invasion dramatically.	[63,71,72,73]
*TFPI2*	Upregulated in young age estrogen receptor-positive breast cancer patients (≤45 years)	Human tissue factor pathway inhibitor-2 (*TFPI-2*) encodes for a wide spectrum of serine proteinase inhibitors which in turn inversely regulates the extracellular matrix degradation, a key step toward tumor invasion and metastasis. *TFPI2* was found to be hypermethylated and consequently downregulated in highly invasive breast cancer cell lines.	[74,75]
*DBX2*	Upregulated in young age estrogen receptor-positive breast cancer patients (≤45 years)	Developing brain homeobox 2 (*DBX2*) plays vital role in cell differentiation and is usually upregulated in cancer tissues. *DBX2* was significantly upregulated in hepatocellular carcinoma tissues and found to play major roles in the proliferation and metastasis of hepatocellular carcinoma cells through Shh pathway.	[76]
*KLK5*	Upregulated in young age oestrogen receptor-positive breast cancer patients (≤45 years)	Overexpression of kallikrein gene was significantly associated with premenopausal, lymph node-positive, and estrogen receptor-negative breast tumors. It was also associated with reduced breast cancer patients, disease-free survival, and overall survival. Finally, *KLK5* was considered unfavourable independent prognostic marker for breast cancer.	[77]
*ESR1*	Downregulated in young age estrogen receptor-positive breast cancer patients (≤45 years)	Downregulation of estrogen receptor has been considered a prognostic factor for local recurrence and distant metastasis. Lower expression of *ESR1* was associated with higher tumor grade.	[78]
*CYP4Z1*	Downregulated in young age estrogen receptor-positive breast cancer patients (≤45 years)	Cytochrome P450 (CYP) 4Z1 frequently overexpressed in breast cancer tissue and correlates with high tumor grade and poor prognosis as *CYP4Z1* has been determined to be involved in tumor angiogenesis and tumor cell proliferation.	[79]
*FOXD2*	Downregulated in young age estrogen receptor-positive breast cancer patients (≤45 years)	Upregulation of *FOXD2-AS1* was associated with poor prognosis among breast cancer patients, as it has been found to be involved in breast cancer cell proliferation, migration, and invasion ability.	[80]
*GREB*	Highly expressed in estrogen receptor-positive breast cancer young age patients	Previous literature suggests that growth regulation by estrogen in breast cancer (*GREB*) is involved in the estrogen-induced growth of breast cancer. It is also considered a clinical marker for response to endocrine therapy.	[81]
*PTHLH*	Highly expressed in estrogen receptor-positive breast cancer young age patients	Elevated parathyroid hormone-like hormone (*PTHLH*) production has been found to be associated with metastasis of breast cancer to the bone.	[82]
*HPGD*	Highly expressed in estrogen receptor-positive breast cancer young age patients	Hydroxyprostaglandin dehydrogenase (*HPGD*) has been determined to be correlated with activity of estrogen pathway and possesses tumor suppressive activity. SNP rs8752 in miR-485-5p binding site in *HPGD* gene was found to be associated with high risk of breast cancer.	[83]
*STK6*	Highly expressed in estrogen receptor-positive sporadic breast cancer young age patients	Aurora kinase A is involved in the proliferation, mitotic checkpoint control, and cell growth in breast cancer basal subtype.	[84]
*FGFR1*	Highly expressed in estrogen receptor-positive sporadic breast cancer young age patients	Fibroblast growth factor receptor 1 is associated with poor prognosis in luminal subtype breast cancer patients; also, it was found to induce endocrine therapy resistance.	[85]
*DLG7*	Highly expressed in estrogen receptor-positive sporadic breast cancer young age patients	Disc large homolog 7 had critical role throughout spindle assembly which is important for genomic stability. Upregulation of *DLG7* is associated with worse overall survival in distinct subtypes of colorectal cancer patients.	[86]

**Table 3:** Implication of EOBC deregulated microRNAs to breast cancer.

MicroRNA	Status in EOBC	Implication to Breast Cancer	Reference
miR-9	Upregulated in estrogen receptor-positive tumors in patients with age ≤ 35 years.	Previous literature suggests direct involvement of miRNA-9 in breast cancer metastasis, due to the different levels of expression through different stages of breast cancer. *MTHFD2* is one of the genes targeted by miR-9, which plays a role in cell viability and anti-apoptotic activity.	[93,94]
miR-210	Upregulated in estrogen receptor-positive tumors in patients with age ≤ 35 years.	A known hypoxia-regulated microRNA, which is upregulated in normal and transformed hypoxic cells, miR-210 has been found to be vital during tumor initiation and growth by reducing mitochondrial respiration in the hypoxic microenvironment.	[95,96]
miR-106a	Upregulated in estrogen receptor-positive tumors in patients with age ≤ 35 years.	The upregulation of miR-106a has been proven to induce breast cancer cell proliferation, colony formation, migration, and invasion in vitro. Moreover, miR-106a upregulation significantly reduces breast cancer cell apoptosis and sensitivity to cisplatin.	[97]
miR-106b	Upregulated in estrogen receptor-positive tumors in patients with age ≤ 35 years.	Breast cancer patients frequently had upregulated level of miR-106b in tissue. miR-106b expression significantly correlated with breast cancer tumor size and marker of proliferation Ki67 expression. High miR-106b expression was associated with shorter disease-free survival and overall survival in breast cancer patients.	[98]
miR-18b	Upregulated in estrogen receptor-positive tumors in patients with age ≤ 35 years.	miR-18b is involved in the modulation of breast cancer cell migration and metastasis in vitro.	[99]
miR-33b	Upregulated in estrogen receptor-positive tumors in patients with age ≤ 35 years.	miR-33b is frequently downregulated in breast cancer tissue in comparison to normal adjacent tissue. A previous study recognized miR-33b as a negative regulator for metastasis and breast cancer cell stemness.	[100]
miR-518a-3p	Upregulated in estrogen receptor-positive tumors in patients with age ≤ 35 years.	Expression of miR-518a-3p was associated with cell proliferation in several malignancies. miR-518a-3p expression was positively correlated with overall survival in triple-negative breast cancer patients.	[101]
miR-372	Downregulated in estrogen receptor-positive tumors in patients with age ≤ 35 years.	Frequently overexpressed in breast tumor tissue. Downregulation of miR-372 significantly inhibited cell proliferation and induced apoptosis of breast cancer cells.	[102]
hsa-miR-1228*	Deregulated in young age breast cancer patients (≤ 35 years).	miR-1228 plays a vital role in the regulation of cell proliferation and metastasis in cancer cells.	[103,104]
hsa-miR-3196	Deregulated in young age breast cancer patients (≤ 35 years).	Few studies elucidate the role of hsa-miR-3196 in cancer progression. However, miR-3196 was downregulated in basal cell carcinoma compared with non-lesional skin.	[105]
hsa-miR-1275	Deregulated in young age breast cancer patients (≤35 years).	Upregulated miR-1275 has been reported to induce p53 signaling pathway via regulating serpin family E member 1(SERPINE1) (which is member in P53 signalling pathway and is also a known protein involved in cell adhesion) that suppresses tumour cells proliferation, invasion, and migration while at the same time, promoting cell apoptosis. (In vitro and in vivo study in glioma). Another role for hsa-miR-1275 is to inhibit adipogenesis in obesity.	[106,107,108]
hsa-miR-1207-5p	Deregulated in young age breast cancer patients (≤35 years).	Low expression of 1207-5p targeted mRNA was reported to regulate tumor protein p53, transforming growth factor β (TGF-b) and insulin signaling pathways in metabolic syndrome patients. P53, TGF-b, and insulin signaling pathways are implicated in the initiation, progression, and metastasis of breast cancer.	[109]
hsa-miR-92b	Deregulated in young age breast cancer patients (≤35 years).	Has-miR-92b expression inhibits breast cancer cell viability, invasion, migration, and control autophagy through histone methyltransferase enhancer of zeste homolog 2 (EZH2) in vitro.	[110]
hsa-miR-139-5p	Deregulated in young age breast cancer patients (≤35 years).	hsa-miR-139-5p expression induces apoptosis and arrests cell cycle in S phase. Furthermore, it inhibits viability, migration, and invasion in breast cancer cells via targeting Notch signaling pathway.	[111]
miR-1285-5p	Correlated with overall survival in young age breast cancer patients (≤35 years).	miR-1285-5p suppresses breast cancer cell proliferation activity via upregulation of targeted gene transmembrane protein 194A (TMEM194A).	[112]
miR-183-5p	Correlated with overall survival in young age breast cancer patients (≤35 years).	miR-183-5p have oncomiR effects on breast cancer cells. Overexpression of miR-183-5p can significantly induce breast cancer cell proliferation and inhibit apoptosis in vitro.	[113]
miR-194-5p	Correlated with overall survival in young age breast cancer patients (≤35 years).	miR-194-5p were found to be upregulated in breast cancer tissue. Knockdown of miR-194-5p in breast cancer cell resulted in inhibition of cell proliferation, migration, and invasion via expression of *SOX17* and regulation of the canonical Wnt (Wnt/β-catenin) signaling pathway.	[114]

**Table 4:** The role of EOBC over- and underexpressed proteins in breast cancer.

Protein	Expression Status in EOBC	Role of Protein	Reference
BCL2L1	Overexpressed	Belongs to the BCL2 protein family that act as anti-apoptotic regulators that are involved in a wide variety of cellular activities. Tamoxifen treatments induce apoptosis by reducing BCL2 level in breast cancer.	https://www.ncbi.nlm.nih.gov/gene/598 (accessed on 4 March 2021), [120]
PARP1	Overexpressed	Co-expressed with other DNA repair proteins suggesting that it may have a role in DNA repair process. PARP1 expression was determined to be related to younger age, larger tumor size, and higher tumor grade in breast cancer.	[121]
RAF1	Overexpressed	Raf-1 and Bcl-2 were found to induce different and overlapping pathways that contributed to drug resistance. Mutations like amplification of c-erbB-2 oncogene, which is frequent in young age breast cancer patients, may lead to activating Raf, resulting in resistance to certain chemotherapeutic drugs.	[122]
ESR1	Low expression	ESR1 lower expression is a predictive factor for endocrine therapy resistance.	[123]
EIF4E	Low expression	eIF4E expression was positively associated with breast tumor grade.	[124]
STAT5A	Low expression	Low level of Stat5a expression was associated with poor prognosis in node-negative breast cancer tumours.	[125]
RPS6KA1	Low expression	RPS6K encodes protein that contributes to the control of cell growth and proliferation.	https://www.ncbi.nlm.nih.gov/gene/6194 (accessed on 4 March 2021)
TFF1	Highly expressed in oestrogen receptor-positive and HER-2-negative breast cancer patients with age (27–56 years)	Expression of TFF1 regulated by estrogen plays a role in breast cell metastasis. Inhibition of TFF1 may increase hormonal therapies’ efficacy.	[126]
RANKL	Highly expressed in oestrogen receptor-positive and HER-2-negative breast cancer patients with age (27–56 years)	RANKL participates in mammary gland development during pregnancy and is involved in the initiation and progression of progesterone-induced breast cancer.	[127]
PgR	Highly expressed in oestrogen receptor-positive and HER-2-negative breast cancer patients with age (27–56 years)	Progesterone receptor plays an important role in breast cancer initiation and progression. Additionally, progesterone receptor expression is important in the prediction and prognosis of breast cancer.	[128]

**Table 5:** Implications of EOBC-deregulated stromal genes in breast cancer.

Stromal Gene	Expression Status in Early-Onset Breast Cancer	Role	Reference
*UQCRQ*	Overexpressed	*UQCRC1* is frequently highly expressed in breast cancer tissues. Expression of *UQCRC1* was positively correlated with cytochrome c-oxidase (COXII), suggesting that it may play a role in cell apoptosis.	[138,139]
*ALDH1A3*	Overexpressed	Aldehyde dehydrogenase (*ALDH1A3*) has been found to be positively correlated with breast cancer tumor grade, stage, and metastasis.	[140]
*EGLN1*	Overexpressed	*EGlN1* (also called the oxygen sensor *PHD2*) is the main prolyl hydroxylase that regulates hypoxia-inducible factor and epidermal growth factor expression in breast cancer. It has been established that breast cancer with high epidermal growth factor level is more aggressive.	[141]
*IGF1*	Overexpressed	In addition to being reported as a potent mitogen for breast cancer cells, *IGF1* and *IGF2* promote proliferation and inhibit apoptosis of breast cancer cells. Knockdown of their expression results in tumor growth inhibition.	[142]
*FUT9*	Underexpressed	Fucosylation is a type of glycosylation process that has been detected excessively in several malignancies. Overexpression of fucosylated antigens was associated with proliferation, metastasis, epithelial mesenchymal transition, and multi-drug resistance.	[143]
*PDHX*	Underexpressed	Reduction in pyruvate dehydrogenase protein X level in cell has resulted in a decrease in mitochondrial oxidation, increase in extracellular acidification, and change in the level of pyruvate, lactate, and citrate. Collectively, this resulted in increasing cell proliferation ability.	[144]
*CCL18*	Underexpressed	Chemokine (C-C motif) ligand 18 (CCL18) is mainly produced by tumor-associated macrophages and is associated with metastasis and poor prognosis in breast cancer patients.	[145]

## Data Availability

Not applicable.

## Review Criteria

We have explored search engines such as PubMed, Google Scholar, and ResearchGate using the keywords as follows: breast cancer, young women, genetic background, differential gene expression, copy variant number, breast cancer pathways, breast cancer mutations, and EOBC. Good-quality articles published in English and Spanish language were included if the data were relevant and useful for our review.
